# Higher Dental Ethics

**Published:** 1895-09

**Authors:** J. H. Smyser


ARTICLE IV.

### HIGHER DENTAL ETHICS.

BY J. H. SMYSER, D. D. S.

It is not my purpose in this brief paper to discuss ethical studies at any great length. It is not necessary to do so for the purpose in hand.

The doctrine of ethics is not an exact science, not even a science for all, for it lacks some of the elements that enter into the definition of the word science.

The ground work upon which the superstructure of science is built is a series of indisputable facts capable of demonstration and easy classification.

The ground work of ethics is a series of propositions almost universally accepted as truth, but which are nevertheless subjected to much specious modifications, no tyro would ever attempt this upon a fact of science.

A sufficient number of propositions or principles of ethics are universally accepted by enlightened society to form a firm basis for the doctrine of ethics, and the highest and noblest branch of speculative phisosophy.

Few, indeed, are the men who would attempt to controvert any part or parcel of the doctrine contained in the simple injunction, "Do unto others as you would have others do unto you." This, of course, is only one of the many index fingers which points with an unerring directness toward the highway along which move the highest type of the upright man.

But I apprehend that the difficulty does lie in the non-acceptance by professional men of the principles of ethics. Their absolute truth finds lodgment in every cultured mind. Their acceptance is practically universal.

There is no room for the ubiquitous liberal to stand on beyond and outside the ethical platform, as to whether he shall believe or not.

The field of controversy lies somewhere else, and it is certainly not hard to find; its crags and peaks loom up somewhere in the hazy distance at many, if not all, of our meetings. There is room for suspicion that the field of doubt sometimes, through a mental upheaval, is thrust up to and into our private offices. A glimpse of the landscape where there is no law but self, and no criterion of success but money getting, is apt to be seductive even if it be illusory.

It is in the practical application of ethical principles to our every-day practice where the "Faith that is in us" is put to the test. This fact is well illustrated, I think, by a discussion between a French professor and a group of his young countrymen. The professor was an instructor on ethics. He had previously attempted to ground his disciples thoroughly in the doctrine contained in the injunction, "Love thy neighbor as thyself." It was review day and he was questioning his class closely in order to test their ability in applying its teachings to practical affairs of life.

The answers of the students came promptly; they showed a high degree of advancement in ethical training; there was no halting, no faltering, no misjudgment. Finally came the question, "How should the French, as a nation, feel toward the Germans, as a nation?" Here was a poser. The wheels of progress were blocked; silence reigned everywhere. It was a case where inclination and plain duty clashed.

Certain images of Alsace and Lorraine began to float before the young Frenchmen, and here began a series of specious modifications and exceptions supposed to free the French from the operations of a plain law, and this picture is a type of the difficulties that beset men everywhere, and in all walks of life. It is the unswerving application of plain rules to the practical affairs of life whatever our own inclinations are, that give rise to doubts and difficulties.

Now, we who are not concerned in the affairs of these two great peoples, who have no inborn feeling in the matter, would have no difficulty in applying the rule in this case; for it is a law that ought to be immutable and binding everywhere and at all times.

Many things contributed to the young men's wavering attitude. Probably the professor's own example, being a historic Frenchman, in his daily life may have been a silent commentator on the doctrine he was enforcing so well in his lecture. It is supposable that his supporting anti-German sentiment, though his political and other association may have had its influence on the minds of his followers. Who knows?

In order to avoid the many undesirable infractions upon the code of ethics we must endeavor to improve our institutions of learning; to do this, more time and attention should be devoted and greater care observed in making good honest impressions upon the student, affecting the character of the professional life he is about to lead.

In passing through many of the colleges of our cities and observing their way of doing things, the relation between instructor, demonstrator and student, and the manner of treating their patients, reminds one of a typical *dental parlor* and the more time devoted to the investigation of those infirmaries the more striking becomes this comparison.

It is the belief of the writer that the old adage, "As the twig is bent so the tree is inclined," can in no place be better applied than right here.

It is not true that in art, business and the professions students follow the general style of their instructors, or the institutions from which they secured their education. We believe that the greater fault lies in the fact that most of our so-called colleges are conducted in too great a measure as money-making mediums, and since, as a rule the men on the higher rounds of the ladder in the profession are the ones usually connected with those institutions, either as directors or faculty, are responsible for the way in which that institution is conducted.

To this fact is due the reason that so little is being done to improve the condition and raise the standard of education, which is the only way by which we can reach the fulfillment of a desirable code of ethics.

To the same fact may be added the inability to obtain any satisfactory legislation in the way of giving authority to corporations to grant license to individuals to practise dentistry. Hence we have weak colleges scattered all over the land, offering any and all kinds of inducements to students to go there and study dentistry, regardless of ability character or previous conditions of servitude. It is not even necessary to know or understand the language used in the institution. The one thing needful is a certain amount of time and money and a specified amount of operative and mechanical work (representing so much cash). Having filled these few most important requirements, they are furnished with license to practise dentistry. What can we expect from such a condition of things, and who is to blame?

I hope each one here to-night will become interested enough in this subject to formulate some answer for himself. A few of the colleges are, without a doubt attempting to improve these conditions, endeavoring to impress upon the students the fact that they have some honesty of purpose, and aside from giving them the best possible instructions upon the theory and practice of dentistry are also inculcating principles of a higher character, satisfying the requirements of the honorable practitioner.

Advertising in its various forms seems to be the leading evil, to which men resort to lower the standing of the profession. It is not necessary to enumerate the different forms used by each individual, since unlike conditions and circumstances demanded in a style peculiar and suitable in each case to make up the deficiency. All alike obnoxious and dishonorable, the main object being to deceive. Whether through the public press, programmes, or gay streamers hung up in public places, they all have the same purpose in view, and the same unprofessional effect in the end. The higher the standing of individuals the greater the harm done.

Take as an example a student at college, writing out the time for appointment with a patron on a card, on the back of which is printed the statement that "We charge for material only," and after the patient returns and has a tooth filled with amalgam, a charge of $1.50 or $2 is made. What a volume of ethical culture goes with such a transaction. What a feeling of honesty there is in it; and then that same student goes down town and drops into one of the hotels, and there on a post he sees the names of the faculty on a silk ribbon, telling where they may be found during certain hours. This, of course, is to convey to the unwary stranger the fact that his name, being on the nice yellow ribbon, would be a more desirable place to receive professional services, than across the way, where the portraits of those many nice looking men with stylishly trimmed whiskers, are placed on a bill board.

No credit or honor in the way of ethical training can ever be claimed by an institution that turns out young men under such conditions, though a few may reach the highest pedestal of fame, and from the time they leave the institution their march is upward and onward, elevating whenever they can, the soiled mantle of our code, by honesty and integrity. Many are fond of taking a different course, resorting to the various unprofessional ways to conduct their business, and in many cases they are only following out the principles taught them in their early education. Other influences might have produced different results.

What I have written upon this subject may exclude a few of the better colleges, but the fact remains that there are many and most of the colleges whose teaching and influence have a lowering instead of an elevating effect upon those brought in contact with them.

I am a firm believer in the saying "That the sins of omission is as great as the sins of commission." We should reach the highest perfection in our better colleges and wipe out of existence those which are conducted only for pecuniary considerations and not for dental education, and when this is done our attention should be called to those disreputable places called "dental parlors," so numerous in all our large cities, some of them being conducted by men who are absolutely destitute of any knowledge upon the subject, have no thought of the high character of their work, the importance of their best skill, but are simply conducted as a business enterprize. Men whose influences are needed to remove these evils, never stoop from their exalted positions to consider the harm done to the profession they are apparently so much endeavoring to elevate. Months of education is required to offset the evil influence of a day, upon those naturally weak in the doctrine of ethics, and much individual, honest effort is necessary to remove the distrust of the public mind.

If it were not a fact that the eyes of the public were kept in darkness concerning the happenings of those places owing to the bountiful revenue contributed to the daily press they could not possibly exist.

Think of the health department interfering with the prayers of Dr. Dowie on ethical exceptions, and allowing such dental parlors to flourish unmolested. Here is an opportunity for some one to promote our ethics, and at the same time become a benefactor.

Abuses are not alone confined to this lower strata of the profession. A common evil among the "upper crust" is in daily practice in the form of exorbitant fees.

The evil in this respect is greater and more dishonorable than in the case of the dental parlor man, from the fact that the class of men in question are of a different type, whose intelligence, and knowledge of values, tell him that the transaction with his dentist was dishonest and is a means of breeding distrust, in destroying the ethical relation between the public and profession, which is of far more importance than that between men of the same profession.

The result of experiences of the above nature can be seen when individuals take up the daily papers and scan the columes for rates on dentistry, pass from the sublime to the ridiculous as it were, take chances with the fates in preference to submitting to an unjust fee.

I do not believe much in technical ethics; different writers upon this subject, draw the line of demarcation between professions too sharply; as, in the case of the city dentist accusing the country physician of a breach of ethics because he extracted an aching tooth for his neighbor; or, on the other hand, the physician censuring the the dentist for advising a remedy for the cure of sick headache. or wiping a chunk of soot from a patient's eye. This is not what is meant by higher ethics, but finds its application in the rules and regulations of labor unions. We should be far beyond any such petty jealousies, place our aim higher, show to the world that we are public benefactors, be honest with each other in all our dealings and carry out to the letter what is implied by the Golden Rule.—*Dominion Dental Journal*

